# High Therapeutic and Esthetic Properties of Extracellular Vesicles Produced from the Stem Cells and Their Spheroids Cultured from Ocular Surgery-Derived Waste Orbicularis Oculi Muscle Tissues

**DOI:** 10.3390/antiox10081292

**Published:** 2021-08-16

**Authors:** Kyung Min Lim, Ahmed Abdal Dayem, Yujin Choi, Yoonjoo Lee, Jongyub An, Minchan Gil, Soobin Lee, Hee Jeong Kwak, Balachandar Vellingirl, Hyun Jin Shin, Ssang-Goo Cho

**Affiliations:** 1 Molecular & Cellular Reprogramming Center (MCRC), Department of Stem Cell & Regenerative Biotechnology, Konkuk University, 120 Neungdong-ro, Gwangjin-gu, Seoul 05029, Korea; lmin0217@konkuk.ac.kr (K.M.L.); ahmed_morsy86@yahoo.com (A.A.D.); trikk33@naver.com (Y.C.); leyjo97@gmail.com (Y.L.); delrar@naver.com (J.A.); minchangil@gmail.com (M.G.); soobineey@naver.com (S.L.); h_jeong9581@naver.com (H.J.K.); 2 Human Molecular Cytogenetics and Stem Cell Laboratory, Department of Human Genetics and Molecular Biology, Bharathiar University, Coimbatore 641-046, India; geneticbala@buc.edu.in; 3 Department of Ophthalmology, Research Institute of Medical Science, Konkuk University Medical Center, Konkuk University School of Medicine, Seoul 05029, Korea

**Keywords:** waste orbicularis oculi muscle tissue, OOM-SC extracellular vesicles, anti-senescence, antioxidant, whitening, wound healing

## Abstract

Extracellular vesicles (EVs) are paracrine factors that mediate stem cell therapeutics. We aimed at evaluating the possible therapeutic and esthetic applications of EVs prepared from the waste human facial tissue-derived orbicularis oculi muscle stem cells (OOM-SCs). OOM-SCs were isolated from the ocular tissues (from elders and youngsters) after upper eyelid blepharoplasty or epiblepharon surgeries. EVs were prepared from the OOM-SCs (OOM-SC-EVs) and their three-dimensional spheroids. OOM-SCs showed a spindle-like morphology with trilineage differentiation capacity, positive expression of CD105, CD 90, and CD73, and negative expression of CD45 and CD34, and their stem cell properties were compared with other adult mesenchymal stem cells. OOM-SC-EVs showed a high inhibitory effect on melanin synthesis in B16F10 cells by blocking tyrosinase activity. OOM-SC-EVs treatment led to a significant attenuation of senescence-associated changes, a decrease in reactive oxygen species generation, and an upregulation of antioxidant genes. We demonstrated the regeneration activity of OOM-SC-EVs in in vitro wound healing of normal human dermal fibroblasts and upregulation of anti-wrinkle-related genes and confirmed the therapeutic potential of OOM-SC-EVs in the healing of the in vivo wound model. Our study provides promising therapeutic and esthetic applications of OOM-SC-EVs, which can be obtained from the ocular surgery-derived waste human facial tissues.

## 1. Introduction

Eyelid blepharoplasty is one of the most commonly performed facial cosmetic procedures [1], and myocutaneous flaps of the orbicularis oculi muscle (OOM) have previously been used to repair facial skin defects of the cheek, nose, and eyelid [2,3]. During these surgeries, however, large amounts of OOM were removed and considered as medical waste. Thus, in this study, we aimed to exploit these waste tissues for the isolation of stem cells with beneficial therapeutic applications.

OOMs, which are rich in vascular distribution, may have regenerative capacity and play vital roles in tissue grafting and facial burn injury repair [4]. Recent reports have shown the efficacy of the ocular muscle tissue as a rich source of stem cells with high proliferation, differentiation, and therapeutic capacities [5,6,7,8,9]. In this regard, OOM-derived stem cells (OOM-SCs) have garnered much attention and application recently for disease therapy and tissue regeneration due to their self-renewal, proliferation, and trilineage differentiation capacities [10].

Extracellular vesicles (EVs) are nano-sized vesicles (approximately 30–150 nm) which play vital roles in cell-cell communication, transportation of key molecules between cells, and maintenance of homeostasis [11,12,13,14,15,16,17]. Few studies have shown the possible clinical applications of stem cell-derived EVs in the treatment of various diseases [18,19,20,21]. EVs and secretomes are components of paramount importance in mesenchymal stem cell (MSC)-associated paracrine action and subsequent regenerative capacities [22,23,24,25]. It is of note that various reports have shown the implications of MSC-derived EVs in tissue regeneration [26,27,28]. Applications of EVs in disease therapy provide several merits over the parent cells, including the ease of handling and storing at −70 °C for a long time, the enhanced binding capacity with the target cells, the reduced immune rejection and tumor formation, the ease of sterilizing via filtration before injection, and the convenience of handling EVs during therapy because of, for instance, easy to control EVs dose and injection route [29,30,31,32,33,34].

EVs are considered as the fingerprints of the original parent cells due to the dependence of their composition and properties on the composition and characteristics of the producing parent cells and could indicate their biological functions in the regeneration of target tissues or organs [35,36,37]. Regardless of the parent cell source, EV-associated markers, which depend on the presence or absence of specific proteins, could determine their purity and unique characteristics [35,36].

EVs possess specific proteins and microRNA (miRNA), which are implicated in EV-associated biological functions [38,39,40]. Moreover, EV-associated lipids are involved in EV-mediated anti-cancer activity and particular lipids possess angiogenic, mitogenic, migratory, and immunomodulatory activities that are consequently useful for healing incurable skin wounds [41,42]. It is of note that the capacity of human MSCs (hMSCs)-derived EVs in the healing of skin wounds is attributed to their robust involvement in regulating cellular proliferation, extracellular matrix remodeling, inflammatory events, angiogenesis, and activation of specific signaling pathways [27,43,44,45,46]. Various reports have proved the crosslink between EVs and the modulation of pathophysiological events in the skin [34,47].

**Figure 1:**
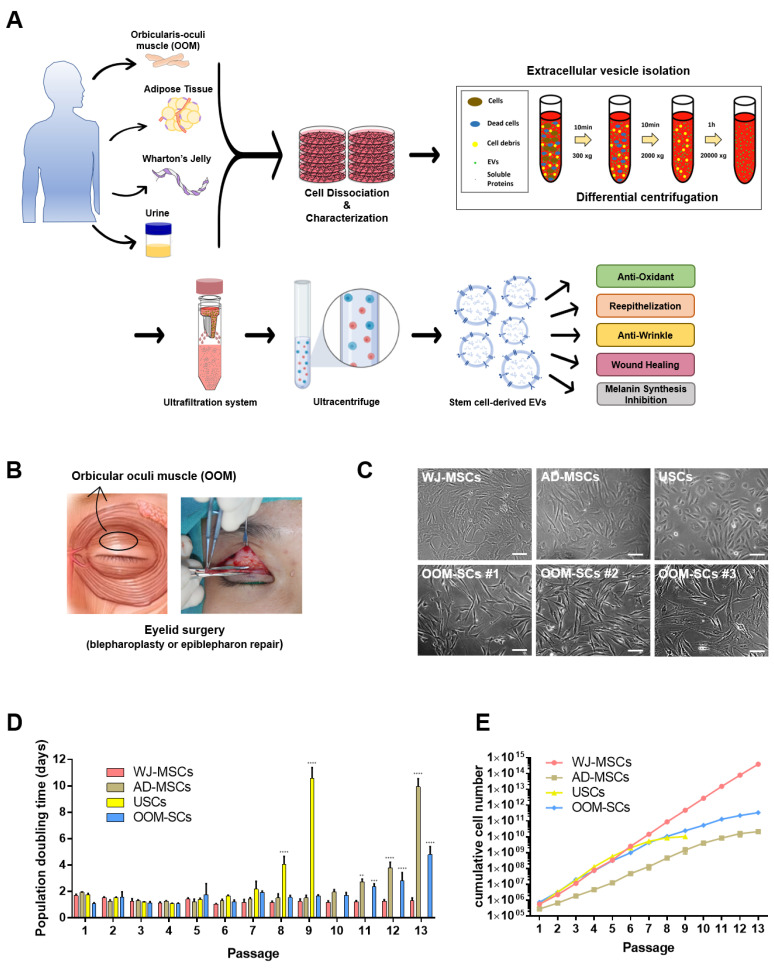
Schematic representation for application of novel OOM-SC-EVs and OOM-SCs preparation. (**A**) Schematic representation of isolation of stem cells from various sources, such as OOM, adipose tissue, Wharton’s Jelly, and urine and then their characterization, which were carried out via the enzymatic digestion using collagenase type II. USCs were obtained via centrifugation at 400× *g*, and purification of EVs from OOM-SCs and WJ-MSCs using differential centrifugation, ultrafiltration, and ultracentrifugation. Our study sought the therapeutic applications of the purified EVs including antioxidant, anti-senescence, whitening, and wound healing. OOM-SC-EVs were isolated from the collected culture soup of the OOM-SCs cultured in α-MEM medium containing exosome-depleted 10% FBS for two days, which were subjected to differential centrifugation, vacuum filtration, ultrafiltration, and ultracentrifugation for EVs purification. The antioxidant, anti-wrinkling, skin whitening, and in vitro and in vivo wound healing activities of the OOM-SC-EVs were observed. These vesicles can be further applied for therapeutic applications in combination with microneedles. Parts of the schematic diagram are created with BioRender.com. (**B**) Figure illustrating the upper eyelid blepharoplasty for the isolation of pretarsal OOM after the consent of donors. OOM-SCs were prepared from healthy donors. (**C**) Morphologies of WJ-MSCs, AD-MSCs, USCs, and OOM-SCs (isolated from different donors (OOM-SCs #1(5y), OOM-SCs#2 (19y), and OOM-SCs#3 (67y)). Phase-contrast microscopic image of isolated cells that showing spindle-like morphologies. Scale bar = 50 μm. OOM-SC proliferation kinetics including (**D**) population doubling time and (**E**) Cumulative cell number of WJ-MSCs, AD-MSCs, USCs, and OOM-SCs. Cell population doubling time was measured up to passage 13. For cumulative cell number, cells were counted using a hemocytometer after staining with 0.4% trypan blue under a phase contrast microscope. Figure 1D data are presented as mean ± SD. All experiments were performed for three independent times, and statistical significance was determined using RMANOVA: ** *p* < 0.01, *** *p* < 0.001, and **** *p* < 0.0001.

In the present study, we examined the characteristics of OOM-SCs in terms of expression of surface markers, stemness markers expression level, colony-forming, and trilineage differentiation capacities in comparison to other MSCs such as human umbilical cord Wharton’s jelly-MSCs (WJ-MSCs), human adipose-derived MSCs (AD-MSCs), and human urine-derived stem cells (USCs). Then, we tried to investigate the therapeutic and esthetic applications of OOM-SC-derived EVs (OOM-SC-EVs) that were purified from OOM-SCs, which were isolated from the ocular surgery-derived waste of human facial tissues from both youngsters and elders. Our results may indicate the possible applications of OOM-SC-EVs for esthetic and reconstructive purposes, which are outlined in Figure 1A.

## 2. Materials and Methods

### 2.1. Preparation and Culture of OOM-SCs, WJ-MSCs, AD-MSCs, and USCs

The experimental protocols for isolation of OOMs and orbicularis oculi fats (OOFs) were approved by the ethics committees of Konkuk University Medical Center (IRB number: KUMC 2019-05-043) and conformed to the principles outlined in the Declaration of Helsinki. For the preparation of OOM-SCs and OOF-derived cells (OOFCs), 16 donors who underwent eyelid blepharoplasty were enrolled in this study; age ranged from 5-year-old to 81-year-old males and females and is listed in detail in supplementary Table 1. All patients provided written informed consent and did not present any other ocular/orbital pathology (dermatochalasis, entropion, and epiblepharon). Muscle fragments were obtained from the pretarsal portion of the OOM during the surgery. Human OOMs and OOFs were washed twice with phosphate-buffered saline (PBS) containing 50 μg/mL gentamicin, and the clotted blood was removed to avoid tissue contamination and blood clots. The tissues were then chopped with a sterile blade and incubated with 4 mg/mL of collagenase type II (Worthington, LS004176) at 37 °C for 90 min with intermittent shaking every 5 min to obtain single cells. After the end of the incubation period, we centrifuged the digested tissues twice at 400× *g* for 10 min to remove the supernatant containing the remaining collagenase. We then suspended the tissue pellets with the alpha-minimum essential medium (α-MEM) (Gibco, Gaithersburg, MD, USA) supplemented with 10% fetal bovine serum (FBS, Hyclone, Logan, UT, USA) and 1% penicillin/streptomycin (PS, Gibco), seeded onto 0.2% gelatin-coated culture dish, and incubated at 37 °C in 5% CO_2_ with the continuous microscopic observation of cell adherence and morphology.

For the preparation of WJ-MSCs, a human umbilical cord was procured from Konkuk University Hospital after approval by the IRB (7001355-201705-BR-181) of Konkuk University. The tissues were chopped and the cells from the tissues were cultured in α-MEM supplemented with 10% FBS and 1% PS. The isolation and characterization of AD-MSCs and USCs were carried out as described in our recent publication [48].

### 2.2. Cell Growth Kinetics, Viability and Colony Formation Assays

Cell growth kinetics were estimated according to population doubling time (PDT) and cumulative cell number. Briefly, cells were seeded at the density of 1 × 10^5^ cells onto a 60 × 15 mm culture plate (SPL, 20060, Pocheon-si, Gyeonggi-do, Korea) and incubated at 37 °C and 5% CO_2_ until reaching 80–90% confluence. Cells were harvested after each passage (P), stained with 0.4% trypan blue solution, and counted using a hemocytometer under a phase-contrast microscope. Cell population doubling was calculated as reported previously [49] and according to the following equation: No(PD) = (log Nt − log N0)/0.301, where PD represents population doubling, Nt denotes cell number after trypsinization/collection of cells, and N0 indicates the number of seeded cells.

For estimation the effect of EVs in the viability of B16F10 melanoma cells, cells were seeded at 3 × 10^3^ cells/well onto a 96-well plate (SPL, 30096) and incubated at 37 °C and 5% CO_2_ overnight. Cells were then exposed to OOM-SC-EVs in a dose-dependent manner for 24 h. Afterward, cells were treated with 10 μL of CCK-8 solution/well (Dojindo, CK04-05, Rockville, MD, USA) and then incubated for 2 h with protection from light. The absorbance was measured at the wavelength of 450 nm using a Bio-RAD x-MarkTM spectrophotometer (Bio-Rad Laboratories, Hercules, California, USA). To measure the self-renewal ability of the stem cells, colony-forming units (CFU) were assessed. Stem cells were seeded at 1 × 10^3^ cells per well in 6-well plates (SPL, 32006) and incubated at 37 °C and 5% CO_2_ for 14 days. Following incubation, the cells were washed and stained with 0.15% crystal violet solution. After washing with PBS, we captured images of the colonies using a microscope (Carl Zeiss).

### 2.3. Reverse Transcription Polymerase Chain Reaction (RT-PCR) and Quantitiative PCR (qPCR) Analyses

Total RNA was isolated from the cells using Labozol Reagent (LaboPass, CMRZ001, Yuseong-gu, Daejeon, Korea) according to the manufacturer’s instructions, and the quantification of the purified RNA was performed using a NanoDrop spectrophotometer (Nanodrop Technologies Inc., Wilmington, DE, USA). cDNA synthesis was performed from 2 μg total RNA using the M-MuLV reverse transcription kit (Labopass, CMRT010) and oligo dT primers. PCR was performed using rTaq Plus 5× PCR Master Mix (ELPISBIOTECH, EBT-1319, Seo-gu, Daejeon, Korea), and PCR products were visualized with 1–2% agarose gels. We performed quantitative real-time PCR via the estimation of the expression changes using the EzAmp™ qPCR 2× Master Mix (ELPISBIOTECH, EBT-1802) and an Applied Biosystems 7500 real-time PCR system. We then normalized the expression level of target genes to the housekeeping gene glyceraldehyde 3-phosphate dehydrogenase (GAPDH), and the relative expression was calculated based on the comparative 2^(−^^ΔΔCt)^ method as reported [50]. The primer sequences for the genes analyzed in this study are shown in Table 1.

### 2.4. Flow Cytometry

Immunophenotyping analysis of OOM-SCs was performed using flow cytometry. Briefly, cells were trypsinized to obtain a single cell suspension and bound with the following primary and secondary antibodies for 10 min on ice. The primary antibodies were used against the following: CD34 (R&D system, Minneapolis, MN, MAB72271), CD45 PD7/26/16 + 2B11 (Invitrogen, Waltham, MA, USA, MA5-13197), CD73/NT5E (Invitrogen, RG235718), CD90/Thy1 (R&D system, AF2067), CD105 (Invitrogen, MA5-11854).

We incubated 1 × 10^9^ particles with CD9 magnetic beads (human exosome-Human CD9 flow detection reagent with Dynabeads^®^ magnetic separation technology; Invitrogen, 10620D) overnight at 4 °C for flow cytometry measurement. Next, the mixture was subjected to brief centrifugation that was followed by washing before using the measurement with the flow cytometry. The antibodies used in the flow cytometry analysis were against the following: CD63-PE (BD Pharmingen, 556020), and CD81-APC (Miltenyi Biotec B.V. & Co. KG, Bergisch Gladbach, Germany, 130-119-787). A flow cytometer (BD Bioscience, San Jose, CA, USA) was used to measure the fluorescence intensity generated by the fluorescent labeled antibody.

### 2.5. Trilineage Differentiation

The trilineage differentiation capacities of the isolated stem cells were estimated via the induction of osteogenic, chondrogenic, and adipogenic differentiation. For osteogenic induction, cells were seeded at 5 × 10^4^ cells/well in 24-well plates and exposed for two weeks to the osteogenic differentiation medium containing 10% Dulbecco’s Modified Eagle Medium (DMEM) low glucose medium supplemented with 50 μg/mL L-ascorbic acid 2-phosphate (Sigma, St. Louis, MI, USA), 10 nM β-glycerophosphate (Sigma), and 100 nM dexamethasone (Sigma). Osteogenic capacity was measured using Alizarin Red S staining (Sigma) to visualize calcium mineralization.

For chondrogenic differentiation, cells were seeded at a density of 5 × 10^4^ cells/well in a 24-well plate and exposed to induction medium, which was composed of 10% DMEM low glucose medium containing 100 nM dexamethasone, 10 nM β-glycerophosphate, 50 μg/mL L-ascorbic acid 2-phosphate, 10 μg/mL transforming growth factor-β1 (Sigma), 1 mM sodium pyruvate (Sigma), 40 μg/mL proline, and 1× insulin-transferrin-selenium (Sigma). The chondrogenic differentiation capacity was evaluated via Alcian Blue staining (Sigma).

For adipogenic differentiation, cells were seeded at 5 × 10^4^ cells/well in a 24-well plate and then exposed to adipogenic differentiation medium containing 10% DMEM low glucose medium supplemented with 10 μg/mL insulin, 500 μM isobutylmethylxanthine (Sigma), 100 μM indomethacin (Sigma), and 1 μM dexamethasone. The adipogenic differentiation capacity was evaluated using Oil Red O staining (Sigma) to visualize the fat droplets.

### 2.6. OOM-SC-EV and WJ-MSC-EV Isolation and Characterization

For the isolation of EVs, OOM-SCs or WJ-MSCs were seeded at 1 × 10^6^ onto 150-mm cell culture dishes with α-MEM medium containing 10% FBS for two days. After reaching 80–90% confluence, we changed the media using a 10% EV-depleted FBS medium, in which the exosome was depleted as described previously [51] for EV isolation. To prepare the EV-depleted FBS, the FBS was subjected to ultracentrifugation at 120,000× *g* for 18 h (Optima L-90K ultracentrifuge, SW32.1 rotor, k-factor 229, Beckman Coulter, Indianapolis, IN, USA). During all the in vitro evaluation experiments of stem cell-derived EVs using various cell lines, we changed the basic culture media to 10% EV-depleted FBS medium that is used for EVs treatment. The culture medium was collected and subjected to differential centrifugation at 300× *g* for 10 min for cell depletion. Subsequently, the supernatant was carefully transferred to a new tube and centrifuged at 2000× *g* for 10 min to remove cell debris. We then transferred the supernatant to a new tube and centrifuged it at 20,000× *g* for 1 h. Subsequently, the resultant supernatant was subjected to filtration using a 0.22-μm vacuum filter (EMD Millipore SCGP00525 Steriflip-GP Filter), and the subsequent filtrate was subjected to ultrafiltration with an Equilibrate Amicon^®^ Ultra-15 filter (#UFC901024, 10 kDa MWCO). Finally, EVs pellets prepared via ultracentrifugation (100,000× *g*, for 2 h) were suspended in 100 µL PBS.

The size of the EVs was determined using dynamic light scattering (DLS) analysis using a Nano Zetasizer (Malvern Instruments, Malvern, UK), and the number of EVs was counted with a nanoparticle tracking analyzer NS300 (NTA, Nanosight, Amesbury, UK) using the following settings: number of captures: four, capture duration: 30 s, Screen gain: 12, camera level: 11, threshold: two, and temperature: 25 °C.

Further, the morphology and structure of EVs were analyzed by transmission electron microscopy (TEM) at 80 kV (JEM-1010, Nippon Denshi, Tokyo, Japan). We then measured the protein concentration of OOM-SC-EVs using a BCA protein assay kit (Pierce, Waltham, MA, USA) according to the manufacturer’s protocol. We used 3 µg of OOM-SC-EVs protein for western blotting analysis for EV-associated markers.

### 2.7. Western Blotting Assay

We measured the protein levels of the isolated EVs using the BCA protein assay kit according to the manufacturer’s protocol. We extracted the cellular proteins using a buffer composed of 100 mM Tris-HCl (pH 7.5), 1% Triton X-100 (Sigma), 10 mM NaCl, 10% glycerol (Amresco, Solon, OH, USA), 50 mM sodium fluoride (Sigma), 1 mM phenylmethylsulfonyl fluoride (PMSF; Sigma), 1 mM p-nitrophenyl phosphate (Sigma), and 1 mM sodium orthovanadate (Sigma). The cell lysates were then centrifuged at 13,000 rpm for 15 min at 4 °C. The supernatant was carefully transferred into a new E-tube, and protein quantification was performed using a BCA protein assay kit. The proteins (3 μg) were separated by 8–12% sodium dodecyl sulfate polyacrylamide gel electrophoresis (SDS-PAGE) and then transferred onto nitrocellulose membranes (Bio-Rad). Membranes were blocked using 5% skimmed milk in Tris-buffered saline for 1 h, followed by incubation overnight at 4 °C with the appropriate primary antibodies (Abs): anti-CD9 (Abcam, ab263023), anti-CD63 (Invitrogen, 10628D), anti-CD81 (Santa Cruz, sc-7637), anti-calnexin (CST, 2679T), anti-GM130 (CST, 12480S), and anti-β-actin (CST, 4970S). Subsequently, the membranes were incubated with secondary antibodies (anti-mouse or -rabbit IgGs), which were conjugated with horseradish peroxidase (Santa Cruz Biotechnology) for 1 h at room temperature. Protein signals were visualized using an enhanced chemiluminescence kit (Amersham Biosciences, Piscataway, NJ, USA) and ChemiDocTM Imaging System (Bio-RAD, 17001401).

### 2.8. Three-Dimensional (3D) Spheroids Culture and EVs Isolation

For 3D culture, OOM-SCs and WJ-MSCs were seeded at 2 × 10^6^ cells per well onto F127 (P2443, Sigma)-coated AggreWell 400^TM^ plates (STEMCELL Technologies, 34425, Vancouver, BC, Canada) and then incubated overnight for embryonic bodies (EBs) formation. Then, we transferred EBs into a 100 mm petri dish (SPL, 10090) and cultured them with a 10% EV-depleted FBS medium. Next, the culture plates were transferred onto an Orbital shaker (ORS) with a sticky mat (INFORS HT Celltron, 69455) and subjected to rotation at 60 rpm for two days. We then collected the media and purified EVs as described above. The characterization of the isolated EVs was performed using NTA as described above. In this experiment, we compared the concentration of 3D culture-derived EVs with monolayer-derived EVs.

### 2.9. Senescence-Associated β-Galactosidase (SA-βgal) Assay

To estimate the impact of OOM-SC-EVs in ameliorating senescence-related changes in the late stage of normal human dermal fibroblasts (NHDFs) (P15–17) (Promocell, C-12302, Heidelberg, Germany), we performed SA-βgal staining as described previously [52]. In brief, cells were seeded onto 6-well plates and incubated at 37 °C and 5% CO_2_ until reaching 80% confluence. Next, cells were exposed to 1.5 × 10^9^ particles/mL of OOM-SC-EVs for 24 h at 37 °C. Cells were then washed with PBS and fixed with a mixture of 0.2% glutaraldehyde (*vol*/*vol*) and 2% formaldehyde (*vol*/*vol*) that was diluted in PBS buffer for 5 min at RT, followed by the removal of the fixative solution and washing twice with PBS. Subsequently, we added a freshly prepared SA-βgal staining solution to the cells and incubated it overnight at 37 °C (without CO_2_). We prepared SA-βgal staining solution using a mixture of 40 mM citric acid/Na phosphate buffer, 5 mM K_3_[Fe (CN)_6_], 5 mM K_4_[Fe (CN)_6_], 3H_2_O, 2 mM magnesium chloride, 150 mM sodium chloride, and 1 mg/mL X-gal dissolved in distilled water. Stained cells were then washed twice with PBS and once with methanol. Finally, cells were dried and kept at room temperature under protection from light until being photographed using phase-contrast microscopy. SA-βgal-positive cells showed blue color.

### 2.10. Reactive Oxygen Species (ROS) Generation Measurement

To measure the modulation of intracellular generation level of ROS after EVs treatment, we measured the changes in the intracellular ROS level using 2′,7′-dichlorodihydrofluorescein diacetate (H_2_DCFDA, Invitrogen) according to the manufacturer’s protocol. In brief, the cells were incubated with 1.5 × 10^9^ particles/mL of OOM-SC-EVs for 24 h at 37 °C. Following incubation, cells were washed twice with PBS and then incubated with 10 μM H_2_DCFDA solution for 30 min at 37 °C and 5% CO_2_ under protection from light by wrapping in aluminum foil. ROS-induced green fluorescent signals were visualized using a fluorescence microscope (Nikon Eclipse TE2000-E). In addition, the fluorescence intensity of the H2DCFDA probe was measured using flow cytometry.

### 2.11. Melanin Content Assay

B16F10 cells were seeded onto 12-well plates at a density of 4 × 10^4^ cells/well and cultured with RPMI medium containing 10% FBS overnight. Subsequently, 200 nM α-melanocyte-stimulating hormone (α-MSH) (sigma, M4135) and dose-dependent exposure of OOM-SC-EVs (1.5, 3, 9, and 15 × 10^8^ particles/mL) or arbutin (100 μM) (Sigma, A4256) were administered and incubated for 60 h. Further, to measure extracellular melanin, we transferred 100 µL of culture medium to a new 96-well plate, and absorbance was detected at 405 nm wavelength with a Bio-RAD x-MarkTM spectrophotometer. Additionally, for intracellular melanin measurement, each well was washed with PBS and then dissolved with 200 µL of 1 N NaOH for 1 h at 80 °C. Absorbance was then measured at 405 nm. The extracellular and intracellular melanin levels were normalized to the total protein concentration.

### 2.12. Tyrosinase Activity Assay

B16F10 cells were cultured at a density of 4 × 10^4^ cells/well onto 12-well plates for 24 h. Subsequently, each well was treated with 200 nM of α-MSH and OOM-SC-EVs (1.5, 3, 9, and 15 × 10^8^ particles/mL) or arbutin (100 μM) and then incubated for 48 h. Each well was washed with PBS, and the cells were harvested with PBS and suspended in 50 mM of phosphate buffer (pH 6.8) containing 1% Triton X-100. After vortexing, the mixture was incubated at 80 °C for 30 min and then subjected to thawing at room temperature. After centrifugation at 1000× *g* for 10 min, 40 μL of the supernatant and 100 μL of 10 mM L-DOPA (Sigma, 333786) were added to a 96-well plate and incubated at 37 °C for 1 h. Absorbance was then measured at 405 nm using a Bio-RAD x-MarkTM spectrophotometer. Each absorbance value was normalized to that of total protein.

### 2.13. In Vitro Cell Migration (Scratch Assay)

To evaluate the effect of OOM-SC-EVs on the migration of NHDFs, which were seeded at a density of 5 × 10^5^ cells/well onto 6-well plates and cultured in high-glucose DMEM containing 10% FBS and 1% PS until confluency. To stop cell proliferation, 10 μg/mL mitomycin C (M4287, Sigma) was treated for 2 h at 37 °C, and subsequently, we scratched the cells with a 200-μL tip after washing. After treatment with 1.5 × 10^9^ particles/mL OOM-SC-EVs or WJ-MSC-EVs, in vitro wound (scratch) closure was examined, and the evaluation of the size of the wound closure (in triplicate) was performed using TScratch software [53].

### 2.14. In Vivo Wound Healing Assay

To analyze the in vivo wound healing capacity, we used six-week-old BALB/c nude female mice (CAnN.Cg-Foxn1nu/CrljOri SPF/VAF Immunodeficient mice), which were purchased from ORIENT BIO Animal Center (Seongnam-si, Korea). The immunodeficient mice (with innate immunity) used in this study are hairless mice, which obviate the difficulties of using haired mice, such as the inflammation or unexpected wounds from the hair removal process, hindering the wound healing process from hair re-growth, and both mice share similar wound healing stages [54]. The in vivo experiment was performed following the approval from the Institutional Animal Care and Use Committee (IACUC) at Konkuk University (approval no.: KU20132-1). One week before the experiment, the mice were kept in a well-ventilated room with adjusted temperature and humidity and under a 12 h light/12 h dark cycle for appropriate adaptation. The animals were provided with food and water ad libitum. They were divided into two groups as follows: (1) Control wounded group (PBS treated) and (2) OOM-SC-EV-treated group (n = 5 mice in each group). Before creating the in vivo wound, all mice were subjected to anesthesia via the intraperitoneal injection of 60 mg/kg of alfaxalone (Alfaxan; Careside, Gyeonggi-do, Korea). Mice were anesthetized, and wounds were excised using a sterile biopsy punch (diameter of 8 mm; Kai Industries, Tokyo, Japan) to make two full-thickness skin wounds on the back of each of the animals. The mice were then injected subcutaneously at four points around each wound; only PBS (control group) and 1.5 × 10^9^ particles/mL of OOM-SC-EVs were suspended in 30 μL PBS. To protect the wounds from external contamination, they were sealed with silicon (0.5 mm T), Tegaderm tape (1622W), and bandage (DUPOL). We monitored the progress in wound closure using a digital camera. The size of the wounds was measured with a 30-cm ruler and recorded using a camera in a time-dependent manner. In addition, we measured scar formation at 2 weeks post-wounding. On day 14 after cell injection, the mice were sacrificed, and skin tissues around the wounds were sectioned, fixed using 4% paraformaldehyde (PFA, Biosesang, Korea), and dehydrated using various concentrations of alcohol, followed by paraffin embedding. Subsequently, the tissues were cut vertically to the wound surface into 4µm thick tissue sections, and the cut tissues were placed on slides that were pre-coated with poly L-lysine 0.1% *w*/*v* (Sigma, St. Louis, MO, USA). For visualization of tissue lesions and regeneration, the sections were subjected to hematoxylin and eosin staining to evaluate the degree of re-epithelization. In addition, Masson’s trichrome staining was used to estimate the rate of collagen synthesis. To obtain the tissue images, the slides were scanned using a digital slide scanner (3d-histech, H-1141 Budapest, Öv u. 3., Hungary).

### 2.15. Statistical Analysis

All statistical analyses were performed using GraphPad Prism (version 7). All experiments were performed independently at least in triplicate. Data are expressed as means ± SD. *p*-values were calculated using one-way ANOVA or RMANOVA and two-tailed *t* test to determine statistical significance. In all figures, the asterisk symbol indicates statistical significance as follows: * *p* < 0.05; ** *p* < 0.01; *** *p* < 0.001; **** *p* < 0.0001, and ns; not significant.

## 3. Results

### 3.1. OOM-SCs Characterization

To prepare OOM-SCs, we obtained muscle fragments from the pretarsal portion of the OOM of healthy patients (both elders and youngsters) who underwent eyelid surgeries. The isolation method for the OOM-derived cells was based on enzymatic digestion using collagenase type II after washing and chopping of the OOM tissues, followed by several centrifugations, and seeding of the obtained single cell onto a 0.2% gelatin-coated culture plate, as shown in Figure 1A. From patients (n = 16) (Supplementary Table S1), we could isolate the pretarsal parts of OOM tissues, which are composed of orbital, preseptal, and pretarsal parts [55] (Figure 1B,C). We also isolated OOFCs using the enzymatic digestion method.

Then, we confirmed the spindle-like morphology and the adherence of OOM-SCs derived from a 74-year-old donor (OOM-SC-74) and the OOFC-74 that derived from the same donor (Figure S1A). We also found a sharp increase in population doubling time in OOFC-74 at P 9, whereas OOM-SC-74 showed an increase in the doubling time at P13 (Figure S1B). OOM-SC-74 showed a marked higher cumulative cell number compared with that shown by OOFC-74 (Figure S1C).

We compared the population doubling time and cumulative number rate of OOM-SCs that isolated from a 5-year-old donor (OOM-SC-5) with that of other MSCs, namely WJ-MSC, AD-MSCs, and USCs. Among the tested MSCs, WJ-MSCs showed the lowest doubling time (30.53 h) and maintained the highest proliferation (3.8 × 10^14^) until P13. It was followed by OOM-SCs that possess a doubling time of 39.58 h and showed the second-best proliferation capacity: (3.3 × 10^11^) up to P13. (Figure 1D,E). ADS-MSCs showed the third-best proliferating MSCs up to P13, whereas USCs maintained the proliferation capacity up to P9 (Figure 1E). The detailed results of cell doubling time and cumulative cell number are described in Table 2.

It is of note that we could not detect any significant difference in the proliferation of OOM-SC-5 or OOM-SC-74. Taken together, OOM-SC-5 showed the best in the cell proliferation kinetics compared with other MSCs except for WJ-MSCs, which showed better proliferation capacity. We, therefore, used WJ-MSCs as a potential control MSCs for our further experiments.

**Figure 2:**
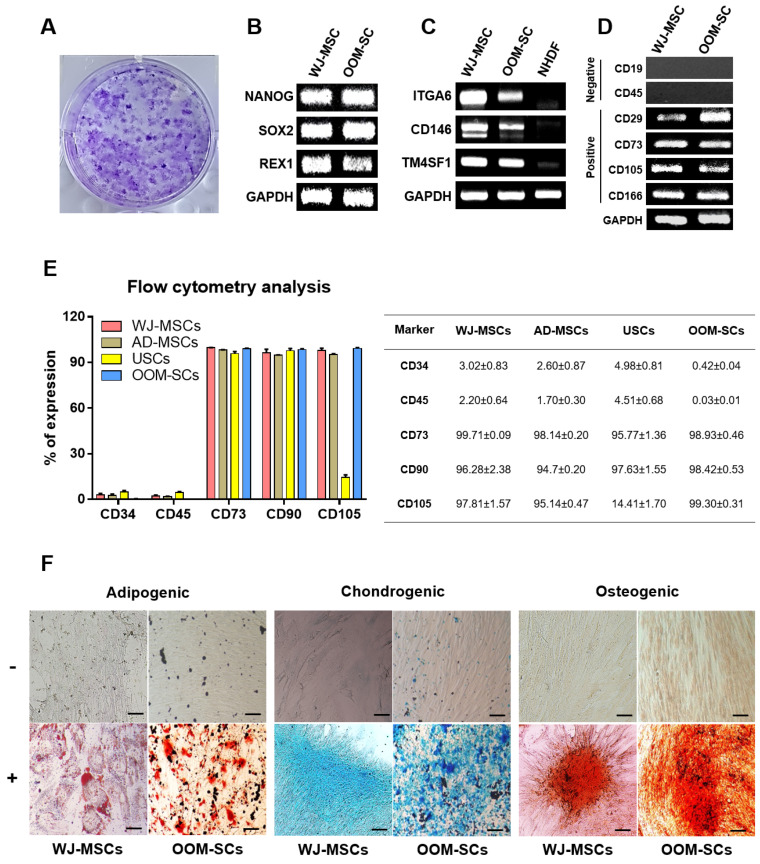
Verification of OOM-SCs Characteristics. (**A**) Microscopic images depict the CFU of OOM-SCs that were visualized with 0.15% crystal violet solution after two weeks of culture. Images of the stained colonies were captured using phase-contrast microscopy. (**B**) RT-PCR results of the expression of the stemness markers Nanog, Sox2, and Rex1 in comparison with those of WJ-MSCs. GAPDH was used as a housekeeping gene. (**C**) RT-PCR results of specific markers, including ITGA6, CD146, and TM4SF1, distinguish between multipotent MSCs and NHDFs. These genes are only expressed in multipotent MSCs. (**D**) RT-PCR data for the expression levels of the surface markers of the isolated OOM-SCs compared with those of WJ-MSCs. These markers included the negatively (CD19 and CD45) and positively (CD73, CD105, and CD166) expressed markers. (**E**) FACS analysis for confirming the expression of the negative markers CD34 and CD45 and positive markers CD73, CD90, and CD105 for OOM-SCs, compared with those of WJ-MSCs. FACS results are summarized in the table (right panel), and all results are presented in percentages. (**F**) Microscopic images of adipogenic, chondrogenic, and osteogenic differentiation were evaluated by Oil Red, Alcian Blue, and Alizarin Red S staining, respectively. The upper and lower panels represent undifferentiated and differentiated cells, respectively. Scale bar = 200 μm.

We found that OOM-SC-5 showed high CFU (Figure 2A). We also verified the high expression of the stemness markers, such as Nanog, Sox2, and Rex1, similar to those of WJ-MSCs (Figure 2B). Notably, both the MSCs and fibroblasts share a similar morphology [56,57,58]; therefore, it is essential to confirm that our cells possess MSC characteristics. The OOM-SC-5 and WJ-MSCs showed apparent expression of integrin subunit α6 (ITGA6), CD146, and Transmembrane 4 L Six Family Member 1 (TM4SF1) genes that are known to be highly expressed in MSCs, although, as expected, NHDFs showed no or weak expression of the genes [59,60,61,62] (Figure 2C). Further, we assessed the expression levels of CD markers to confirm the MSC properties of OOM-SC-5, and we found that these cells significantly expressed CD29, CD73, CD105, and CD166 MSC-positive markers but not CD19 and CD45 MSC-negative markers (Figure 2D), strongly indicating that OOM-SC-5 are a type of MSCs. In addition, we compared the expression levels of CD markers of OOM-SCs with other adults MSCs including, WJ-MSCs, AD-MSCs, and USCs using the flow cytometry analysis. As shown in other adults MSCs, OOM-SC-5 positively express CD73, CD90, and CD105, whereas they negatively express CD34 and CD 45 Figure 2E. It is of note that USCs showed the lowest expression of CD 105 compared with other MSCs and OOM-SCs. The flow cytometry data is presented in percentages in a table (Figure 2E. right panel). We also found that OOM-SC-5 have adipogenic, chondrogenic, and osteogenic differentiation capacities, which were measured using Oil red O, Alizarin red S, and Alcian blue staining, respectively (Figure 2F). The OOM-SC-5 showed similar adipogenic and osteogenic differentiation capacities to those of WJ-MSCs and higher differentiation ability than that of USCs (Figure 2F, Table 3). In the case of chondrogenic differentiation, OOM-SC-5 showed a similar differentiation capacity to that of USCs (Table 3). Taken together, OOM-SC-5 were confirmed to possess the characteristic features of MSCs, most similarly with WJ-MSCs.

### 3.2. OOM-SC-EVs and WJ-MSC-EVs Characterization

**Figure 3:**
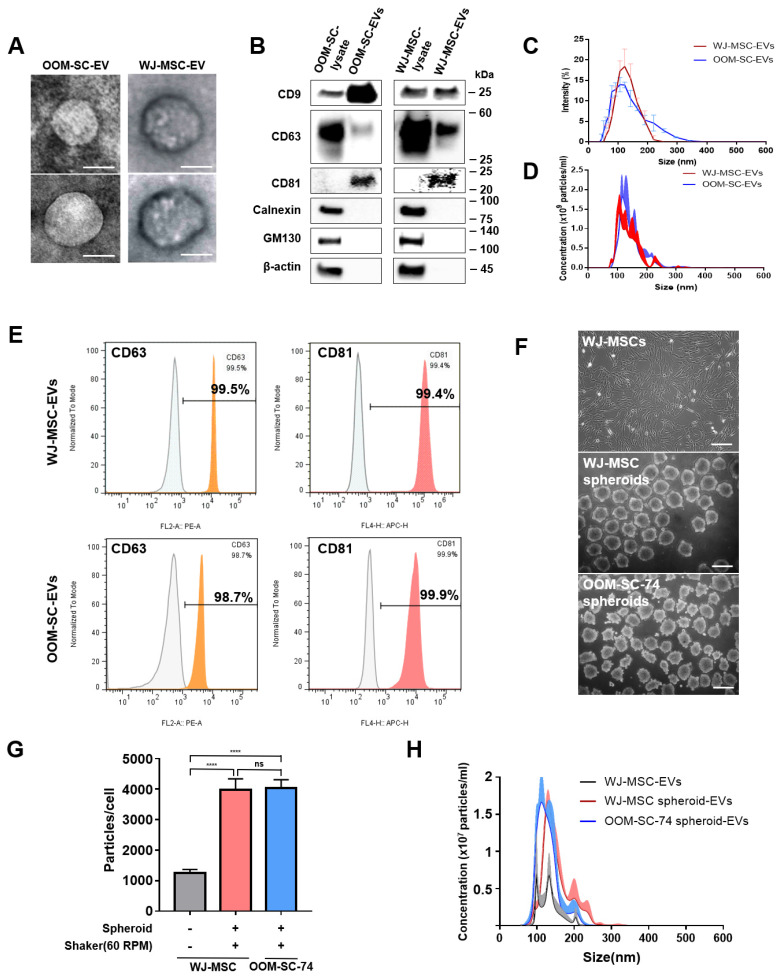
Characterization of OOM-SC-EVs. (**A**) TEM images of EVs showing cup- or sphere-shaped morphology. TEM analysis was performed at 80 kV. Scale bar = 100 nm. (**B**) Characterization of the expression levels of the EV-associated positive surface markers, including CD9, CD63, CD81, calnexin, and GM130, via immunoblotting. We used 3 µg of EVs for the immunoblotting analysis. The protein expression levels of the EV markers were compared with those of the whole cell lysate. (**C**) Dynamic light scattering analysis of EVs sizes. The average diameter of the purified EVs was of a size range of 90 to 120 nm. (**D**) Nanoparticle tracking analyzer NS300 for counting EV numbers. EVs concentrations were 1.66 × 10^10^ particles/mL for OOM-SC-EVs and 1.5 × 10^10^ particles/mL for WJ-MSC-EVs. (**E**) FACS analysis confirmed the expression of EV-associated markers, namely CD63 and CD81. (**F**) Phase-contrast microscopic pictures of WJ-MSCs monolayer and spheroids of WJ-MSCs and OOM-SCs (OOM-SC-74). For spheroid culture, WJ-MSCs and OOM-SCs were seeded onto F127 -coated AggreWell 400^TM^ plates for 24 h for EB formation. Then, EBs were transferred to the ORS at 60 rpm for two days. EVs were isolated from both spheroids and the monolayer simultaneously. Scale bar = 200 μm. (**G**,**H**) Nanoparticle tracking analyzer NS300 for counting EV numbers in monolayer and spheroids. We detected 1246 particles/cell in WJ-MSCs monolayer, whereas EVs yield from WJ-MSCs and OOM-SCs spheroids were 3984 particles/cell and 4033 particles/cell, respectively. Data shown in Figure 3G are presented as mean ± SD. Statistical significance was determined using One-way ANOVA: **** *p* < 0.0001, ns; not significant.

To prepare OOM-SC-EVs and WJ-MSC-EVs, cells were cultured in 10% exosome-depleted FBS media for 48 h until reaching 80–90% confluency. Dead cells and cell debris were removed via differential centrifugation, and the ultrafiltration process, and EVs were collected in a filter device using an Amicon 10-kDa (pore size) regenerated cellulose membrane. The TEM images of the purified EVs revealed the morphology of the purified EVs, which were cup- or sphere-shaped (Figure 3A). We confirmed the expression levels of EV-associated positive markers including the tetraspanin membrane proteins, namely CD9, CD63, and CD81 using immunoblotting (Figure 3B). Moreover, we verified the expression levels of EV-negative markers, such as calnexin and 130-kDa cis-Golgi matrix protein (GM130), which were not detected in the OOM-SC-EVs and WJ-MSC-EVs (Figure 3B).

The EVs size distribution was tracked using a Nanosizer or DLS, and we could not detect any significant variation in the size between OOM-SC-EVs and WJ-MSC-EVs with a size range of 90 to 120 nm (Figure 3C), while their concentration was determined to be 1.66 × 10^10^ particles/mL for OOM-SC-EVs and 1.5 × 10^10^ particles/mL for WJ-MSC-EVs, which measured using NTA (Figure 3D). Finally, we confirmed the expression levels of the positive EVs markers using FACS analysis. For OOM-SC-EVs, CD63, and CD81 expression levels were 99.5% and 99.4%, respectively. In the case of OOM-SC-EVs, the CD63 and CD81 expression levels were 98.7% and 99.9%, respectively (Figure 3E).

In addition, supplementary Table S2 summarizes the criteria for EVs preparation and characterization according to the recommendations of Minimal information for studies of extracellular vesicles 2018 (MISEV2018) that calibrate the EVs preparation procedure [63].

### 3.3. Advanced 3D Culture Approach for Efficient EVs Production

In this experiment, we pursued an efficient approach for EVs production with a high yield in comparison with the monolayer culture system. For that purpose, we prepared spheroids from OOM-SC-74 and WJ-MSCs using ORS at 60 rpm for two days. Figure 3F shows the spheroids morphology of OOM-SC-74 and WJ-MSCs compared with the monolayer morphology of WJ-MSCs. We purified EVs from the culture supernatant of the cultured spheroids and monolayer, and we measured the number of the particles per cell and the EVs concentration. We could isolate a significantly higher particles number per cell (Figure 3G) and higher concentration (Figure 3H) in the case of 3D culture compared with the monolayer culture.

### 3.4. OOM-SC-EV Treatment Modulated ROS Generation and DELAYED Senescence-Related Changes in NHDFs

**Figure 4:**
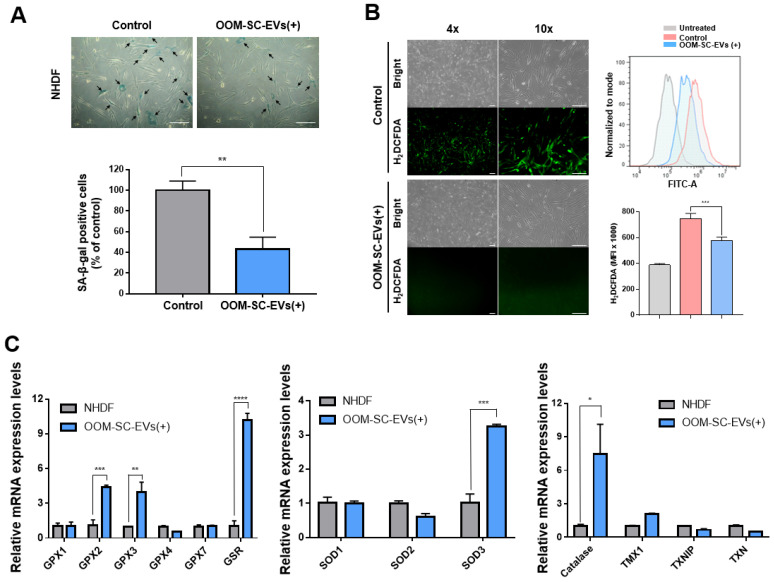
OOM-SC-EVs alleviate senescence-associated changes and modulate ROS generation. (**A**) SA-βgal assay showing a significant reduction of SA-βgal-positive cells after OOM-SC-EV treatment. SA-βgal-positive cells are stained blue. The lower panel graphical data represents the number of SA-βgal-positive cells shown in the upper panel, shown as a percentage of that of the control cells (late passage NHDF). Data are presented as mean ± SD. Statistical significance was determined using Two-tailed *t* test: ** *p* < 0.01. Scale bar = 200 µm. (**B**) Mitigation of ROS generation in OOM-SC-EV-treated late passage NHDF cells. ROS signals were detected via incubation in a 10 μM H2DCFDA solution for 30 min at 37 °C, followed by observation using a fluorescence microscope. The left panel shows the visualization of ROS green signals using the fluorescent microscope, while the right panels show the FACS-mediated quantitative analysis of ROS generation. The left panel shows the visualization of ROS green signals using the fluorescent microscope, while the right panel shows the FACS-mediated quantitative analysis of ROS generation. Data are presented as mean ± SD. Statistical significance was determined using One-way ANOVA: *** *p* < 0.001. Scale bar = 200 μm. (**C**) RT-PCR analysis of the expression levels of antioxidant genes in OOM-SC-EV-treated cells. OOM-SC-EVs significantly increased the expression levels of the antioxidant genes GPX2, GPX3, GSR, SOD3, and catalase. Data are presented as mean ± SD. Statistical significance was determined using Two-tailed *t* test: * *p* < 0.05, ** *p* < 0.01, *** *p* < 0.001, and **** *p* < 0.0001.

We examined the potential of OOM-SC-EVs in delaying senescence-associated changes in NHDFs. The late-stage NHDFs showed a high number of SA-βgal-positive, blue-colored cells; however, treatment with OOM-SC-EVs led to a significant decrease in SA-βgal positive cells compared with control cells (Figure 4A). We then measured the modulation of ROS generation following OOM-SC-EV treatment and found that high levels of ROS generation in late passage NHDFs were significantly suppressed upon the treatment (Figure 4B). Moreover, we observed that this treatment resulted in a significant increase in the expression levels of antioxidant genes, such as glutathione peroxidase (GPX) 2, GPX3, glutathione S-reductase (GSR), and catalase (Figure 4C). Additionally, OOM-SC-EV-exposed cells showed a marked increase in the expression level of extracellular superoxide dismutase (SOD3) (Figure 4C).

### 3.5. OOM-SC-EV Treatment Modulated the Melanin Synthesis of B16F10 Melanoma Cells

**Figure 5:**
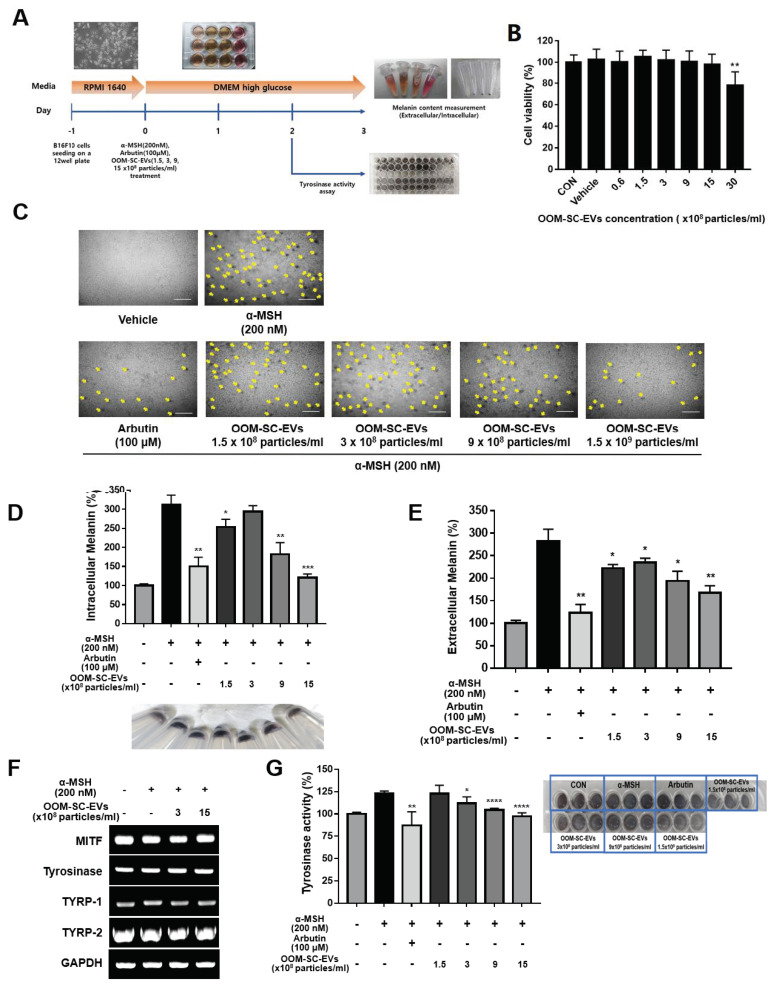
Modulatory effects of OOM-SC-EVs on melanin synthesis and tyrosinase activity. (**A**) A representative scheme elucidates the experimental schedule for evaluating the effects of OOM-SC-EVs on melanin synthesis and tyrosinase activity. In this experiment, we cultured B16F10 melanoma cells using a growth medium (RPMI 1640). The cells were then exposed to OOM-SC-EVs in a dose-dependent manner in combination with arbutin (melanin synthesis inhibitor) and α-MSH (melanin synthesis activator) in DMEM high glucose medium. Tyrosine activity and melanin content were measured after OOM-SC-EV and inhibitor treatments on day 2 and day 3, respectively. (**B**) Effect of concentration-dependent exposure of OOM-SC-EVs on the viability of B16F10 cells. B16F10 cells were plated at 3 × 10^3^ cells/well in a 96-well plate and maintained in serum-free RPMI medium overnight. The cells were then exposed to OOM-SC-EVs in a concentration-dependent manner (0.6, 1.5, 3, 9, 15, and 30 × 10^8^ particles/mL) for 48 h. Subsequently, we added 10 μL of CCK-8 solution/well (Dojindo, CK04-05), followed by incubation for 2 h with protection from light. Absorbance was measured at 450 nm using a Bio-RAD x-MarkTM spectrophotometer. (**C**) Microscopic images showing the dose-dependent inhibitory action of OOM-SC-EVs on α-MSH-mediated melanosome formation compared with that of the melanin synthesis-suppression chemical arbutin. The formation of melanosomes is indicated by a yellow arrow. OOM-SC-EVs showed a dose-dependent suppression of melanosome formation. Scale bar = 100 µm. (**D**) Measurement of intracellular melanin levels after treatment with OOM-SC-EVs. Significant inhibition of melanin synthesis by OOM-SC-EVs was detected at 9 and 15 × 10^8^ particles/mL, similar to the inhibitory effect of arbutin. The lower panel showing the qualitative inhibitory action of OOM-SC-EVs against melanosome formation was visualized in cells via a change in the color of the cell pellets. (**E**) Estimation of the effect of OOM-SC-EVs on the suppression of α-MSH-mediated induction of extracellular melanin levels compared to that of arbutin. (**F**) RT-PCR data for measuring changes in the expression levels of melanin synthesis-associated genes, including tyrosine synthesis-related genes, such as MITF, tyrosinase, TYRP-1, and TYRP-2, after treatment with OOM-SC-EVs in combination with α-MSH. GAPDH was used as a housekeeping gene. (**G**) Measurement of tyrosinase activity after treatment with various concentrations of OOM-SC-EVs compared with those after treatment with arbutin and α-MSH for 48 h. Subsequently, cells were harvested, permeabilized, frozen and thawed, and centrifuged. For the tyrosine activity assay, the supernatant was mixed with 10 mM L-DOPA in a 96-well plate and incubated for 1 h at 37 °C. The activity was measured at 405 nm using a spectrophotometer and represented as a percentage of the control value. The lower panel shows the visual analysis of tyrosinase activity in a 96-well plate. Data shown in Figure 5B,D,E,G are presented as mean ± SD. Statistical significance was determined in Figure 5B,D,E,G using Two-tailed *t* test: * *p* < 0.05, ** *p* < 0.01 *** *p* < 0.001, and **** *p* < 0.0001.

We investigated the effect of OOM-SC-EV treatment on cell viability or melatonin synthesis in B16F10 melanoma cells (Figure 5A). The melanoma cells were seeded onto 12-well plates in RPMI 1640 medium and cultured until confluency. The cells were then cultured in DMEM high glucose media and treated with OOM-SC-EVs. There were no significant changes in cell viability upon treatment with 1.5, 3, 9, and 15 × 10^8^ particles/mL OOM-SC-EVs, although a slight decrease in cell viability was detected upon treatment with 30 × 10^8^ particles/mL OOM-SC-EVs (Figure 5B). Subsequently, we treated the B16F10 melanoma cells with a melanin synthesis activator (α-MSH, 200 nM), melanin synthesis inhibitor (arbutin, 100 μM), or OOM-SC-EVs (1.5, 3, 9, and 15 × 10^8^ particles/mL) and found that OOM-SC-EV treatment led to an apparent inhibitory activity on α-MSH-induced melatonin synthesis in a dose-dependent manner (Figure 5C). Additionally, we measured the intracellular and extracellular melanin contents upon OOM-SC-EV treatment and found that this treatment resulted in significant suppression of both intracellular (cell pellets) and extracellular melanin contents (Figure 5D, E). Further, we evaluated changes in the mRNA expression levels of tyrosine synthesis-related genes, namely microphthalmia-associated transcription factor (MITF), tyrosinase-related protein-1 (TYRP-1), and TYRP-2, and tyrosinase activity upon OOM-SC-EV treatment. Our results showed that although there were no significant changes in the expression of the tyrosine synthesis-related genes (Figure 5F), tyrosinase activity was significantly reduced (Figure 5G). This tyrosinase activity-suppressing effect of OOM-SC-EV treatment was also visually confirmed (Figure 5G lower panel).

### 3.6. In Vitro and In Vivo Wound Healing and Anti-Wrinkle Capacities of OOM-SC-EVs

**Figure 6:**
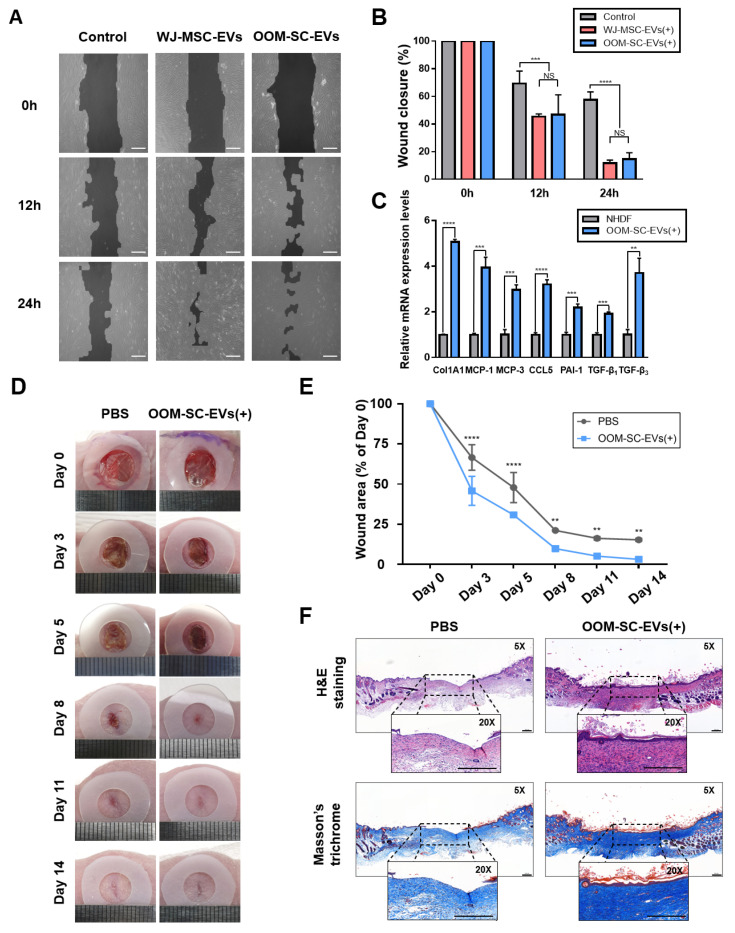
In vitro and in vivo wound healing capacities and the anti-wrinkling activity of the OOM-SC-EVs (**A**) Effect of OOM-SC-EVs in the closure of in vitro scratches of fully confluent NHDFs tablet 10. μg/mL mitomycin C for 2 h and then scratched with a 200-μL tip. Subsequently, cells were exposed to 1.5 × 10^9^ particles of OOM-SC-EVs or WJ-MSC-EVs in a time-dependent manner (0, 12, and 24 h). Scale bar = 200 μm. (**B**) Graphic diagram representing the in vitro scratch assay in Figure 6A, and the wound closure was evaluated using TScratch software. Data are presented as mean ± SD. Statistical significance was determined using RMANOVA with post-hoc analysis: *** *p* < 0.001, and **** *p* < 0.0001, NS; not significant. (**C**) RT-PCR results showing changes in the expression levels of anti-wrinkle-associated genes. OOM-SC-EV treatment markedly increased the expression levels of collagen synthesis-related genes, namely ColA1, MCP-1, MCP-3, CCL-5, PAI-1, TGF-β1, and TGF-β3, which are related to high collagen synthesis and the treatment of wrinkles. Data are presented as mean ± SD. Statistical significance was determined using Two-tailed *t* test: ** *p* < 0.01 *** *p* < 0.001, and **** *p* < 0.0001. (**D**) In vivo wound healing assay. In this model, two full-thickness skin wounds, on the back of each mouse, were excised via a sterile biopsy punch, followed by the subcutaneous injection of 1.5 × 10^9^ particles/mL OOM-SC-EVs diluted in 30 μL PBS. Wound closure was monitored every two days until day 14. (**E**) Graphical data depicting the closure of the wound area as calculated relatively to the original wound area on day 0 after inducing the injury (n = 5) and in a time-dependent manner. Data are presented as mean ± SD. Statistical significance was determined using RMANOVA with post-hoc analysis: ** *p* < 0.01, and **** *p* < 0.0001. (**F**) Hematoxylin and eosin and Masson’s trichome staining of the mice skin after sacrifice (on day 14). Two weeks after injection of OOM-SC-EVs into the experimental wound, the skin tissues were fixed with 4% PFA and then dehydrated using various concentrations of alcohol, followed by paraffin embedding. For the evaluation of regeneration and re-epithelization of the wound after OOM-SC-EV application, the sections were stained with hematoxylin and eosin. Further, Masson’s trichrome staining was performed to estimate collagen synthesis rate. Scale bar = 200 μm.

The wound healing and anti-wrinkle capacities of OOM-SC-EVs were examined. We conducted a cell scratch assay using NHDFs and found that NHDF migration increased upon OOM-SC-EV treatment. OOM-SC-EVs led to a similar NHDFs migration-increasing activity to that of WJ-MSC-EVs (Figure 6A,B). We also measured the effect of OOM-SC-74-EVs and OOFC-74-EVs on the in vitro wound closure, in which OOM-SC-74 -EVs showed a significant closure of the in vitro wound, especially at 24 h post scratch (Figure S2). Moreover, we found that OOM-SC-EVs treatment significantly increased the expression levels of the anti-wrinkle-related genes ColA1, monocyte chemoattractant protein-1 (MCP-1), MCP-3, chemokine (C-C motif) ligand 5 (CCL-5), and plasminogen activator inhibitor-1 (PAI-1), transforming growth factor-β (TGFβ) 1, and TGFβ3 (Figure 6C). It is of note that our data show the increased ratio of TGFβ3 to TGFβ1, which is in agreement with a previous report’s conclusions on the effect of the increased ratio of TGFβ3 to TGFβ1 by MSC derived exosome treatment in efficient in in vivo scarless wound healing [64].

In addition, we confirmed the in vivo wound healing capacity of OOM-SC-EVs using an animal model. After performing a full-thickness skin wound on the mice back, we injected OOM-SC-EVs subcutaneously in the vicinity of the experimental wound and observed a significant wound healing effect (Figure 6D,E). Moreover, we confirmed the marked re-epithelization of the wound following OOM-SC-EVs treatment using hematoxylin and eosin and Masson’s trichrome staining (Figure 6F).

## 4. Discussion

The goal of our research is to efficiently utilize the waste of human facial tissue from the daily performed ocular surgeries for therapeutic (blepharoplasty and ptosis repair) or cosmetic (double lid crease formation) purposes in both elders and youngsters. Eyelid blepharoplasty is a widespread cosmetic surgery, during which the removed OOM tissues are discarded as medical waste. Compared to muscles in other body sites, the OOM in the eyelids is easily accessible under local anesthesia with little discomfort to the patient, and its removal is far from being associated with loss of function and morbidity for the donor due to its high regenerative capacity. The OOM consists of three anatomic regions, including pretarsal, preseptal, and orbital regions, and each region is suggested to have a different physiological function in the histological microstructure [65]. The pretarsal OOM used in this study contained a high ratio of muscle tissue and a low percentage of fat tissue [65].

In this study, we isolated OOM-SCs from the pretarsal OOMs using the enzymatic digestion method. The OOM-SCs successfully adhered to the culture plate and showed a spindle-like morphology, high clonogenic proliferative behavior, and high stemness and self-renewal capacities. Generally, MSCs have various unique features, including trilineage differentiation potential, stemness, and self-renewal capacities [66]. Similarly, compared with MSCs including WJ-MSCs, AD-MSCs, and USCs, the OOM-SCs showed expression profiles of MSC surface markers and trilineage differentiation potential. Moreover, we confirmed the high expression levels of ITGA6, CD146, and TM4SF1, which are specifically expressed in multipotent MSCs but not in fibroblasts.

The therapeutic potential of MSC-derived EVs has been widely reported [67,68,69,70,71,72]. EVs, which are secreted by most cells, are lipid vesicles with small membranes with diameters of 30–120 nm [73,74]. Stem cell-derived EVs have been reported to contain transcription factors that are secreted by stem cells, which are responsible for maintaining stem cell properties, as well as secreted signaling molecules, such as WNTs, TGF-β, and β-catenin, implicated in EV-mediated therapeutic actions [75,76,77]. EVs can also convey RNAs, including mRNAs, miRNAs, and lncRNAs, which represent their cells of origin [78,79,80,81,82,83,84]. Interestingly, EVs possess the capacity to transfer specific proteins and genetic materials to the recipient cells and, also, EV membranes can be engineered using organ-specific therapeutic proteins or drugs for treating the incurable disease of target organs [85,86,87,88,89,90]. Moreover, the application of EVs obviates the biosafety issues incurred by the direct application of whole stem cells, including tumorigenicity and immunogenic reactions [91,92]. In the field of ophthalmology, the capacity of EVs to treat immune-related eye diseases, retinal inflammation, and retinal ischemia and their application as biomarkers for ocular diseases have been reported [93,94,95,96,97]. The in vivo corneal wound healing activity of MSC-derived exosomes has also been demonstrated [98]. Here, we could effectively isolate and characterize OOM-SC-EVs and reveal their high therapeutic effects on melanogenesis suppression, the anti-wrinkle process, and wound healing. According to the guidelines determined by MISEV2018 for EVs nomenclature, the term EVs can be applied to particles that are naturally released from cells and are characterized by their inability to replicate, are surrounded by a lipid bilayer, and lack any functional nucleus [63]. According to the size, EVs could be classified into small EVs (less than 100 nm or 200 nm) and large or medium EVs (more than 200 nm) [63]. In our study, we carried out differential centrifugation, ultrafiltration, and ultracentrifugation to get rid of debris, dead cells, macrovesicles, and most of the soluble proteins. We ultimately obtained small EVs of 90–120 nm.

Interestingly, OOM-SC-EVs led to marked suppression of enhanced ROS generation in the late-passaged (aged) NHDFs. Moreover, they increased the expression levels of antioxidant genes, namely catalase, GPX2, GPX3, GSR, and SOD3 in aged NHDFs. Our data also showed the capacity of OOM-SC-EVs in the in vitro and in vivo wound closure in NHDFs and mice, respectively. This result is consistent with a previous report that umbilical cord MSC-derived EVs showed potent capacities for enhancing wound healing and skin regeneration [39,99]. In addition, OOM-SC-EVs markedly enhanced the expression levels of collagen synthesis-related genes, namely ColA1, MCP-1, MCP-3, CCL-5, PAI-1, TGFβ1, and TGFβ3. Previous reports have demonstrated the capacities of stem cell-derived exosomes in the alleviation of skin aging change [100], enhancement of collagen synthesis, angiogenesis, and scar healing [101,102]. Additionally, EVs have been previously implicated in ROS modulation [103]. To our knowledge, this is the first study revealing the roles of OOM-SC-EVs in the improvement of wound healing in vitro and in vivo and in the significant delay in skin aging by promoting the expression levels of collagen synthesis-related genes. We hypothesized that OOM-SC-EV-induced upregulation of collagen synthesis genes may be one of the mechanisms that mediate efficient wound healing. Further studies are required to confirm the crosslink between OOM-SC-EVs and collagen synthesis and to verify OOM-SC-EV-related ROS modulation in crosslinks with their therapeutic activities. In addition, the role of OOM-SC-EVs in modulating the immune and inflammatory responses during the wound healing process needs in-depth investigations.

Based on our findings, we can postulate the uptake of OOM-SC-EVs into the epidermis and dermis, followed by the interaction of OOM-SC-EVs with fibroblasts. For clinical or esthetic applications, this effect can emulate the application of microneedles, which are microscopic applicators used to deliver vaccines or other drugs across various barriers, while the transdermal application is the most popular use of microneedles. Microneedles may help overcome poor skin penetration of OOM-SC-EVs, and several novel exciting microneedle concepts may be of great help for the intradermal delivery of OOM-SC-EVs in the future [104].

Melanin possesses a protective function against the harmful effects of ultraviolet (UV) light. However, excess melanin synthesis can result in unfavorable skin disorders, such as spots, freckles, skin hyperpigmentation, and melisma [105,106]. Therefore, inhibition of melanin synthesis has garnered attention in the fields of dermatology and cosmetics. In our study, we showed that OOM-SC-EVs treatment significantly suppressed melanin synthesis in B16F10 cells. Although OOM-SC-EVs treatment did not significantly change the expression levels of melanogenesis-related genes, such as MITF, tyrosinase, TYRP-1, and TYRP-2, it resulted in the significant inhibition of tyrosinase activity. We hypothesized that there is another mechanism associated with OOM-SC-EV-mediated inhibition of melanin synthesis. For instance, previous reports have demonstrated the implication of high ROS generation and depletion of antioxidant factors in the overproduction of melanin [107]. Moreover, several reports have revealed the application of antioxidant agents for the treatment of high melanin-mediated hyperpigmentation [108,109]. Here, we demonstrated the antioxidant activity of OOM-SC-EVs, and therefore, we could postulate the role of OOM-SC-EV-related antioxidant activity in melanogenesis inhibition, which needs further investigation.

We sought to improve the yield of OOM-SC-EVs for large scale production in the future, and so we tried to culture OOM-SCs using the 3D platform using the ORS, and we found a marked increase in the EVs yield upon using the 3D platform compared with using 2D culture system (Figure 3). We are planning to perform an in-depth investigation on the mechanism implicated in the high yield of EVs during the 3D culture system compared to the monolayer system in a future study. We also will verify the capacity of the 3D-derived EVs in the disease model. It is noteworthy that we could not detect any significant difference in terms of cell number, EVs yield, and functions between cells obtained from youngsters and elders. Notably, the 3D platform showed a significantly better EVs yield than that shown in the 2D platform (Figure 3). Therefore, in our future study, we planned to devise a detailed efficient protocol for OOM-SC-EVs production using the 3D culture system. Our study affords a good source for stem cell yield that ultimately produces high-quality EVs, which possess various applications including antioxidant, anti-wrinkle, anti-senescence, whitening effect, and skin regenerative capacity (Figure 1A).

## 5. Conclusions

In conclusion, we were able to efficiently isolate and culture OOM-SCs from the human facial tissues (from youngsters and elders) that were discarded after eyelid blepharoplasty or epiblepharon surgeries. Stem cell properties of OOM-SCs were compared with human adult MSCs (WJ-MSCs, AD-MSCs, and USCs), and possible therapeutic applications of OOM-SC-EVs were verified. Our findings provide new insights into various possible therapeutic and esthetic applications of OOM-SC-EVs, including wound healing, modulation of collagen synthesis-related genes, whitening effect, anti-senescence activity, inhibition of tyrosinase activity, and anti-wrinkling activity. Importantly, we exploited the waste human facial tissues from the daily performed ocular surgeries for therapeutic (blepharoplasty and ptosis repair) or cosmetic (double lid crease formation) purposes in both elders and youngsters. Notably, the 3D platform showed a significantly better EVs yield than that shown in the 2D platform. Our study provides promising therapeutic and esthetic applications of OOM-SC-EVs, which can be competently isolated from the waste human facial tissues.

## Figures and Tables

**Table 1:** Primer sequences of the genes used in this study.

Gene (Application)	NCBI Accession Number	Forward Primer (5′ to 3′) Reverse Primer (5′ to 3′)	Product Length (bp)
**NANOG** **(Semi-qPCR)**	NM_001355281.2	TCCTGAACCTCAGCTACAAAC GCGTCACACCATTGCTATTC	108
**SOX2** **(Semi-qPCR)**	NM_003106.4	CATCACCCACAGCAAATGAC GAAGTCCAGGATCTCTCTCATAAA	110
**REX1** **(Semi-qPCR)**	NM_001304358.2	GTTTCGTGTGTCCCTTTCAAG CTGTTATCTGCTTCATCCTGTTG	141
**ITGA6** **(Semi-qPCR)**	NM_001394928.1	CGAAACCAAGGTTCTGAGCCCA CTTGGATCTCCACTGAGGCAGT	151
**CD146** **(Semi-qPCR)**	NM_006500.3	GTGTTGAATCTGTCTTGTGAA ATGCCTCAGATCGATG	600, 419
**TM4SF1** **(Semi-qPCR)**	NM_014220.3	GGCTACTGTGTCATTGTGGCAG ACTCGGACCATGTGGAGGTATC	133
**CD19** **(Semi-qPCR)**	NM_001385732.1	CCTGGGGTCCCAGTCCTATG GCTCCAGAGGTTGGCATCAT	287
**CD45** **(Semi-qPCR)**	NM_080921.4	TCAGTGGTCCCATTGTTGTG GCATCTCTGTGGCCTTAGCT	146
**CD29** **(Semi-qPCR)**	NM_002211.4	GCCGCGCGGAAAAGATG ACATCGTGCAGAAGTAGGCA	206
**CD73** **(Semi-qPCR)**	NM_002526.4	TATCCGGTCGCCCATTGATG ACGCTATGCTCAAAGGCCTT	138
**CD105** **(Semi-qPCR)**	NM_001278138.2	CCAAGACCGGGTCTCAAGAC TGTACCAGAGTGCAGCAGTG	174
**CD166** **(Semi-qPCR)**	NM_001627.4	GAACACGATGAGGCAGACGA CCGAGGTCCTTGTTTACATGTTT	179
**GAPDH** **(Semi-qPCR)**	NM_001357943.2	AATCCCATCACCATCTTCCAG ATGACCCTTTTGGCTCCC	146
**GPX1** **(qPCR)**	NM_001329503.2	CAGTCGGTGTATGCCTTCTCG GAGGGACGCCACATTCTCG	105
**GPX2** **(qPCR)**	NM_002083.4	GGTAGATTTCAATACGTTCCGGG TGACAGTTCTCCTGATGTCCAAA	174
**GPX3** **(qPCR)**	NM_001329790.2	AGAGCCGGGGACAAGAGAA ATTTGCCAGCATACTGCTTGA	153
**GPX4** **(qPCR)**	NM_001367832.1	GAGGCAAGACCGAAGTAAACTAC CCGAACTGGTTACACGGGAA	100
**GPX7** **(qPCR)**	NM_015696.5	CCCACCACTTTAACGTGCTC GGCAAAGCTCTCAATCTCCTT	86
**GSR** **(qPCR)**	NM_001195102.3	TTCCAGAATACCAACGTCAAAGG GTTTTCGGCCAGCAGCTATTG	94
**SOD1** **(qPCR)**	NM_000454.5	GGTGGGCCAAAGGATGAAGAG CCACAAGCCAAACGACTTCC	227
**SOD2** **(qPCR)**	NM_001322816.2	GCTCCGGTTTTGGGGTATCTG GCGTTGATGTGAGGTTCCAG	92
**SOD3** **(qPCR)**	NM_003102.4	ATGCTGGCGCTACTGTGTTC CTCCGCCGAGTCAGAGTTG	99
**Catalase** **(qPCR)**	NM_001752.4	TGTTGCTGGAGAATCGGGTTC TCCCAGTTACCATCTTCTGTGTA	87
**TMX1** **(qPCR)**	NM_030755.5	AGTATGTCAGCACTCTTTCAGC CACACTGGCAATCCAAGGTCT	83
**TXNIP** **(qPCR)**	NM_006472.6	GGTCTTTAACGACCCTGAAAAGG ACACGAGTAACTTCACACACCT	87
**TXN** **(qPCR)**	NM_001244938.2	GTGAAGCAGATCGAGAGCAAG CGTGGCTGAGAAGTCAACTACTA	87
**Col1A1** **(qPCR)**	NM_000088.4	GATTCCCTGGACCTAAAGGTGC AGCCTCTCCATCTTTGCCAGCA	107
**MCP-1** **(qPCR)**	NM_002982.4	AGAATCACCAGCAGCAAGTGTCC TCCTGAACCCACTTCTGCTTGG	98
**MCP-3** **(qPCR)**	NM_006273.4	ACAGAAGGACCACCAGTAGCCA GGTGCTTCATAAAGTCCTGGACC	117
**CCL5** **(qPCR)**	NM_002985.3	CCTGCTGCTTTGCCTACATTGC ACACACTTGGCGGTTCTTTCGG	125
**PAI-1** **(qPCR)**	NM_001386456.1	CTCATCAGCCACTGGAAAGGCA GACTCGTGAAGTCAGCCTGAAAC	154
**TGF-β1** **(qPCR)**	NM_000660.7	TACCTGAACCCGTGTTGCTCTC GTTGCTGAGGTATCGCCAGGAA	122
**TGF-β3** **(qPCR)**	NM_001329939.2	CTAAGCGGAATGAGCAGAGGATC TCTCAACAGCCACTCACGCACA	161
**GAPDH** **(qPCR)**	NM_001357943.2	GTCTCCTCTGACTTCAACAGCG ACCACCCTGTTGCTGTAGCCAA	131

**Table 2:** Comparison of WJ-MSCs, AD-MSCs, USCs, and OOM-SCs in the cumulative cell number and doubling time in correlation with the volume of tissue of origin.

	WJ-MSCs	AD-MSCs	USCs	OOM-SCs
Volume/Length	14 cm	60 cc	100 mL	2 cm
Cumulative Cell Number	3.8 × 10^14^	2.1 × 10^10^	1.0 × 10^10^	3.3 × 10^11^
Doubling time	30.53 h	42.34 h	44.46 h	39.58 h

**Table 3:** Trilineage differentiation capacity of OOM-SCs compared with those of WJ-MSCs and USCs.

	WJ-MSCs	USCs	OOM-SCs
Adipogenic	+++	++	+++
Chondrogenic	+++	++	++
Osteogenic	+++	++	+++

## Data Availability

The data are presented within the paper. Additional raw data are available on request from the corresponding author.

## Supplementary Materials

The following are available online at https://www.mdpi.com/article/10.3390/antiox10081292/s1, Table S1: List of human orbicular oculi muscle and fat tissue samples, Table S2: Checklist of EV isolation and characterization method, Figure S1: Morphology and proliferation kinetics of OOM-SC-74 and OOFC-74, Figure S2: In vitro wound healing assay of OOM-SC-74-EVs and OOFC-74-EVs.
