# Supplementary material for: High Therapeutic and Esthetic Properties of Extracellular Vesicles Produced from the Stem Cells and Their Spheroids Cultured from Ocular Surgery-Derived Waste Orbicularis Oculi Muscle Tissues
Source: Antioxidants (Basel). 2021 Aug 16;10(8):1292. doi: 10.3390/antiox10081292 (PMC8389225; doi:10.3390/antiox10081292)
Supplement: Supplementary file 1 [file antioxidants-10-01292-s001.zip › antioxidants-1272272-Supplementary.pdf]

Supplemental Information

Supplementary table 1. List of human orbicular oculi muscle and fat tissue samples

Gender	Age	Clinical diagnosis	Tissue
Male	63	Dermatochalasis	Muscle
			Fat
Male	61	Dermatochalasis	Muscle
			Fat
Male	67	Dermatochalasis	Muscle
			Fat
Female	76	Dermatochalasis	Muscle
			Fat
Male	5	Epiblepharon	Muscle
Female	81	Dermatochalasis	Muscle
			Fat
Male	47	Dermatochalasis	Muscle
			Fat
Male	69	Dermatochalasis	Muscle
			Fat
Female	74	Dermatochalasis	Muscle
			Fat
Female	70	Dermatochalasis	Muscle
			Fat
Male	64	Dermatochalasis	Muscle
			Fat
Female	71	Dermatochalasis	Muscle
			Fat
Female	59	Dermatochalasis	Muscle
			Fat
Male	19	Entropion	Muscle
			Fat
Male	19	Entropion	Muscle
			Fat
Female	23	Entropion	Muscle (Upper eyelid)
			Muscle (Lower eyelid)

Supplementary table 2. Checklist of EV isolation and characterization method

	Method	Related keywords	Results
Isolation	Ultrafiltration	Filter membrane type, pore size, Centrifugation speed and time	Regenerated cellulose membrane<br>Amicon 10-kDa<br>5000 xg , 30 min
	Ultracentrifuge	rotor(s), adjusted k factor(s), speed and time	Optima L-90K ultracentrifuge, SW32.1 rotor, k-factor 229, 100,000 xg, 2 h
Characterization	Electron microscopy	Morphology	cup- or sphere-shaped morphology.<br>TEM analysis was performed at 80 kV
	Western blot	At least three positive protein markers of EVs<br>At least one negative protein marker	CD9, 63, 81 (Positive)<br>Calnexin, GM130 (Negative)
	DLS / NTA	Concentration, size range	1.66 x 10^{10} particles/mL<br>90 to 120 nm
	Flow cytometry	Surface marker detection	CD9 Bead-based / CD63 and CD81 positive markers

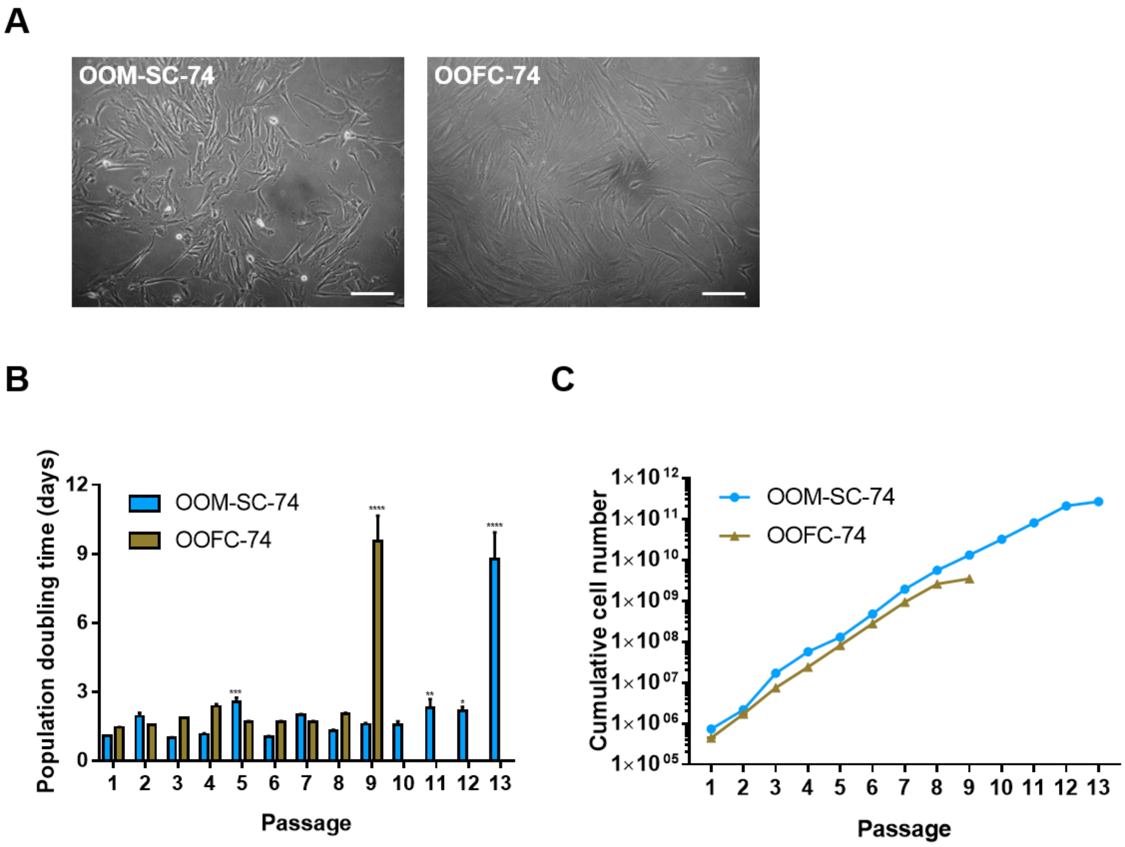

Fig. S1 Morphology and proliferation kinetics of OOM-SC-74 and OOFC-74.

A. Phase contrast microscopic image of isolated OOM-SC-74 and OOFC-74 that isolated form the same donor (74-year-old female) showing a spindle-like morphology. Scale bar=50 µm.

B. Cell population doubling for the OOM-SC-74 and OOFC-74 that measured up to passage 13. For that, cells were counted using a hemocytometer after staining with 0.4% trypan blue under a phase contrast microscope. Statistical significance was determined using RMANOVA: *p<0.05, **p<0.01, ***p<0.001 and ****p<0.0001 (n=3).

C. The cumulative cell number of OOM-SC-74 and OOFC-74 in each passage and up to passage 13. Hemocytometer was used for cell counting.

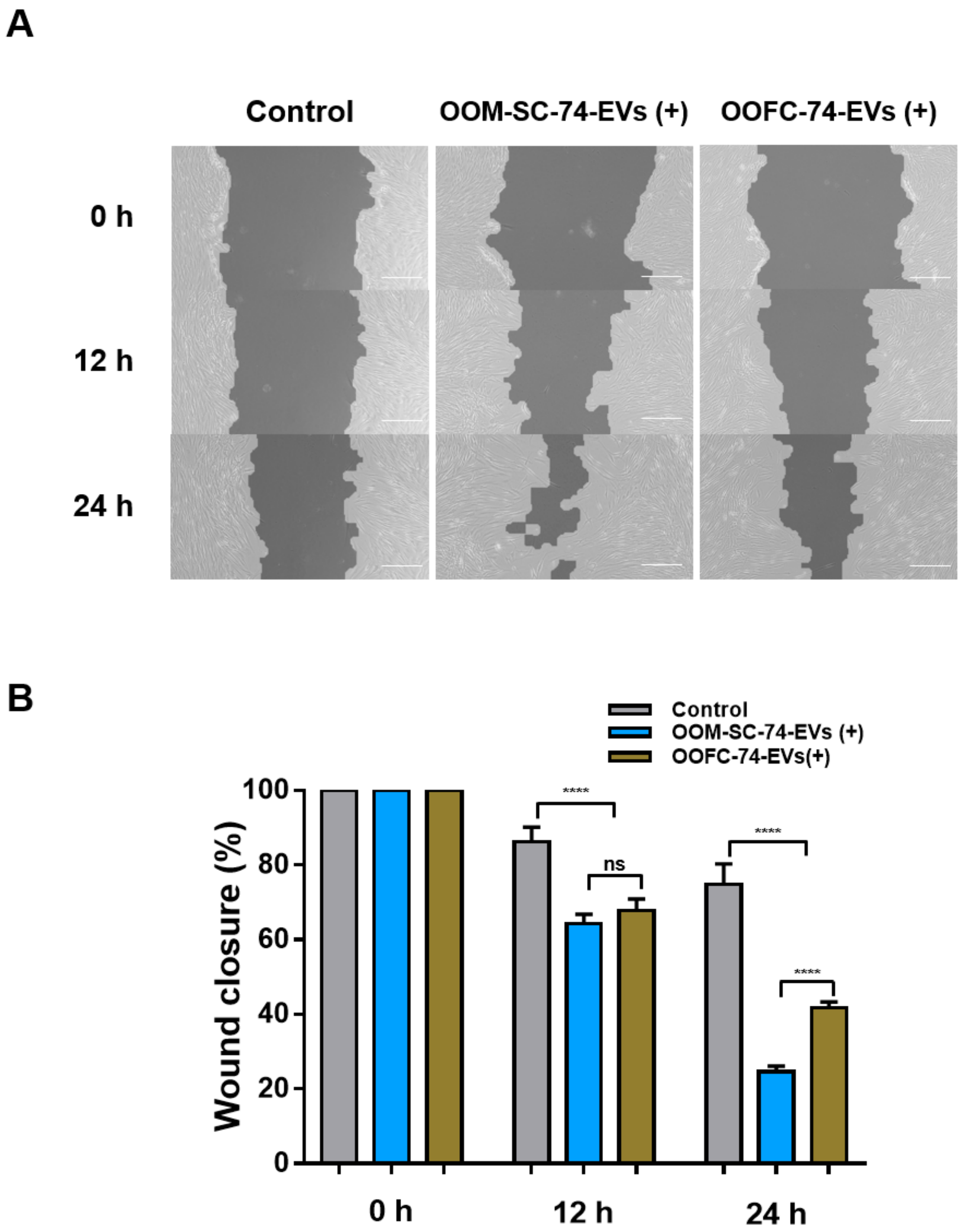

Fig. S2 ***In vitro*** **wound healing assay of OOM-SC-74-EVs and OOFC-74-EVs.**

A. Evaluation the effects of OOM-SC-74-EVs and OOFC-74-EVs in the closure of *in vitro* scratches of NHDFs (fully confluent), which scratched with a 200-μL tip. Subsequently, cells were exposed to 100 μg of OOM-SC-74-EVs and OOFC-74-EVs in a time-dependent manner (0, 12, and 24 h). Scale bar=200 μm.

B. Graphical figure illustrating the *in vitro* scratch assay in Fig. S2A, and the wound closure was measured using TScratch software. Statistical significance was determined using RMANOVA with post-hoc analysis: ****p <0.0001 and ns; not significant (n=3)
